# Hepatocyte Growth Factor Overexpression Slows the Progression of 4NQO-Induced Oral Tumorigenesis

**DOI:** 10.3389/fonc.2021.756479

**Published:** 2021-12-14

**Authors:** Xiaoxi He, Si Chen, Yinghua Tang, Xiaomin Zhao, Liting Yan, Lihong Wu, Zhicong Wu, Weijia Liu, Xinming Chen, Xinhong Wang

**Affiliations:** ^1^ Department of Oral Mucosal Diseases, Affiliated Stomatology Hospital of Guangzhou Medical University, Guangdong Engineering Research Center of Oral Restoration and Reconstruction, Guangzhou Key Laboratory of Basic and Applied Research of Oral Regenerative Medicine, Guangzhou, China; ^2^ Key Laboratory for Oral Biomedical Engineering of the Ministry of Education, Department of Oral Implantology, School and Hospital of Stomatology of Wuhan University, Wuhan, China; ^3^ Department of Periodontics, Affiliated Stomatology Hospital of Guangzhou Medical University, Guangdong Engineering Research Center of Oral Restoration and Reconstruction, Guangzhou Key Laboratory of Basic and Applied Research of Oral Regenerative Medicine, Guangzhou, China; ^4^ Department of Periodontics, Wuxi Stomatology Hospital, Wuxi, China; ^5^ Department of Basic Oral Medicine, Affiliated Stomatology Hospital of Guangzhou Medical University, Guangdong Engineering Research Center of Oral Restoration and Reconstruction, Guangzhou Key Laboratory of Basic and Applied Research of Oral Regenerative Medicine, Guangzhou, China; ^6^ Department of Pathology, School and Hospital of Stomatology of Wuhan University, Wuhan, China

**Keywords:** OSCC, HGF, c-Met, 4NQO, transgenic mouse, tumorigenesis

## Abstract

**Objectives:**

To investigate the role of hepatocyte growth factor (HGF)/c-Met signaling in oral malignant transformation.

**Methods:**

We used immunohistochemistry to investigate HGF and c-Met expression in 53 oral squamous cell carcinoma (OSCC) specimens and 21 adjacent nontumor specimens and evaluated the associations between HGF and c-Met expression and clinicopathological parameters. Additionally, HGF-overexpression transgenic (HGF-Tg) and wild-type (Wt) mice were treated with 4-nitroquinoline-1-oxide (4NQO) to induce oral carcinogenesis for 16 weeks. At 16, 20, and 24 weeks, tongue lesions were collected for clinical observation; estimation of HGF, c-Met, and PCNA expression; apoptosis (TUNEL) assays; and RNA sequencing (RNA-seq).

**Results:**

HGF and c-Met were positively expressed in 92.5% and 64% of OSCC samples, respectively. High HGF expression was significantly associated with smaller tumor size (p = 0.006) and inferior TNM stage (p = 0.032). No correlation between HGF and c-Met levels and other clinical parameters or prognosis was noted. In addition, HGF and c-Met expression was elevated in 4NQO-induced lesions of Wt mice. Compared with Wt mice, HGF-Tg mice have lower tumor incidence, number, volume, and lesion grade. In addition, the percentage of PCNA-positive cells in Wt mice was significantly higher than that in HGF-Tg mice at different time points. At 16 weeks, HGF-Tg mice exhibited less apoptotic cells compared with Wt mice (p < 0.000), and these levels gradually increased until the levels were greater than that of Wt mice at 24 weeks (p < 0.000). RNA-seq data revealed that 140 genes were upregulated and 137 genes were downregulated in HGF-Tg mice. KEGG enrichment analysis showed that upregulated differentially expressed genes (DEGs) are highly correlated with oxidative and metabolic signaling and that downregulated DEGs are related to MAPK and PI3K-AKT signaling.

**Conclusions:**

HGF and c-Met expression is upregulated in OSCC tissues and is associated with the occurrence and development of OSCC. HGF overexpression in normal oral epithelial tissue can inhibit 4NQO-induced tumorigenesis potentially through inhibiting proliferation and accelerating apoptosis *via* MAPK and PI3K-AKT signaling.

## Introduction

Oral squamous cell carcinoma (OSCC) is one of the most common malignant neoplasms with a high morbidity and mortality (1, 2). The development of OSCC is a multistep process that requires the accumulation of multiple genetic changes as a consequence of a patient’s genetic predisposition and long-term exposure to carcinogens, including tobacco, cigarette smoke, and alcohol (3). The features of aggressive invasion and high metastasis and recurrence rate lead to a poor prognosis for OSCC (4). Despite advances in current combination therapy, including surgery, radiotherapy, and chemotherapy, the 5-year survival rate remains less than 50%, and the patient’s quality of life is seriously affected (5). However, early detection and diagnosis followed by appropriate treatment can greatly improve survival. Over the past few decades, numerous studies have been devoted to the discovery of promising tumor molecular markers to help the diagnosis, classification, prognosis, and treatment of cancer.

Hepatocyte growth factor (HGF) is a multifunctional cytokine secreted primarily by mesenchymal cells that contributes to cell proliferation, motility, survival, and morphogenesis (6, 7). HGF binding to mesenchymal–epithelial transition factor (c-Met), known as its unique high-affinity receptor and expressed on epithelial cells, plays an important role in embryogenesis, organogenesis, tissue repair, and wound healing (8, 9). However, studies have confirmed that the HGF/c-Met signaling pathway is abnormally activated in various types of cancer, such as lung cancer, breast cancer, gastric cancer, and head and neck squamous cell carcinoma (HNSCC), and is involved in the oncogenesis, invasion, and angiogenesis of tumors (10, 11). Therefore, HGF/c-Met signaling has become one of the most popular targets for tumor treatment. A variety of targeted drugs targeting this signaling pathway have been developed, and most of them have entered the stage of preclinical research and clinical trials (12). Seiwert et al. showed that the proportion of HGF-positive expression was 59%, and Met overexpression was noted in greater than 80% of 97 HNSCC tissues (13). In addition, serum HGF levels in HNSCC patients were significantly greater than that in healthy people and declined after initial treatment (14). Several studies have suggested that HGF/c-Met signaling is related to OSCC progression (15, 16). *In vitro*, HGF promoted migration and invasion, whereas c-Met inhibitors inhibited migration and viability and promoted apoptosis in OSCC cell lines by activating AKT, ERK1/2, and NF-ĸB signaling (17).

However, recent studies have found that the HGF/c-Met pathway plays a multifunctional role in regulating physiological and pathological processes, including cancer. Finisguerra et al. demonstrated that HGF/c-Met-dependent nitric oxide released by neutrophils can kill tumor cells and protect the tissues around tumors, inhibiting tumor growth and metastasis. However, this signaling pathway can promote the development of Met-addicted tumors, indicating that the HGF/c-Met pathway plays a double-edged role in cancer (18). Moreover, Met deficiency in immune cells can promote tumor progression, and the therapeutic benefit of Met kinase inhibitors is partly weakened by the inhibition of antitumor neutrophils, which require HGF/c-Met activation. Additionally, Dong et al. recently discovered that pulp stem cells overexpressing HGF have dual effects in rheumatoid arthritis (RA). In the early stage before RA induction, HGF inhibited the progression of RA due to its immunosuppressive effect. However, in the late stage, HGF promoted synovitis by producing pathogenic interleukin-6 (IL-6), accelerated proliferation and induced apoptosis resistance (19). Therefore, the HGF/c-Met pathway may have different effects at different stages of disease development.

Previous studies explored the function and role of HGF/c-Met in OSCC mainly through *in vitro* cell experiments. Moreover, they observed the effects of HGF/c-Met signaling on the proliferation, metastasis, and angiogenesis of OSCC cell lines *in vitro*; however, only a few studies have explored the function and role of this pathway in carcinogenesis that refers to the transformation of a normal cell into a tumoral neoplastic cell. Abnormally activated HGF/c-Met signaling in OSCC can promote the development of tumor cells. However, according to the latest research hypothesis, its role in the stage of oral carcinogenesis may be different.

In this study, we aimed to explore the role of HGF/c-Met signaling in the malignant stage of the oral mucosal epithelium by establishing tongue cancer models of HGF transgenic mice (HGF-Tg) and wild-type (Wt) mice induced by chemical carcinogens (4NQO). In addition, we examined HGF and c-Met expression in 53 OSCC tissues and *evaluated the correlation between HGF and c-Met expression and clinicopathological parameters*.

## Materials and Methods

### Patients

This study was approved by the human ethics research committee of Stomatological Hospital Affiliated to Guangzhou Medical University (KY2019025), and informed consent was obtained from all participants. In total, 74 paraffin-embedded tissue samples, including 53 OSCC tissues and 21 adjacent nontumor tissues from 2002 to 2006, with complete clinicopathological data of patients were provided by Stomatological Hospital Affiliated to Wuhan University. Follow-up ranged from 3 to 99 months. These patients had no other malignancies in the 5-year period before therapy, and none received treatment including chemotherapy and radiotherapy before surgery or biopsy. In addition, distant metastasis was not noted in any case. The recorded clinicopathologic parameters included sex, age, tumor size, tumor site, tumor grade, lymph node metastasis, and TNM stage. TNM staging of OSCC was determined by two pathologists according to the eighth edition of the AJCC Cancer Staging Manual. The overall survival (OS) value was from the date of first diagnosis (by biopsy or surgery) to the time of death or the last follow-up. The detailed clinical information of the patients is listed in **Table 1**.

**Table 1:** Association of HGF and Met expression with clinicopathologic parameters in OSCC patients.

Clinicopathologic parameters	No.	HGF expression		*P1*	Met expression		*P2*
		low	high		low	high	
Gender				0.899			0.774
Male	29	8	21		17	12	
Female	24	7	17		15	9	
Age (years)				0.831			0.533
<60	33	9	24		21	12	
≥60	20	6	14		11	9	
Tumour site				0.403			0.453
Tongue	40	13	27		23	17	
Non-tongue	13	2	11		9	4	
Tumour size				0.006**			0.528
≤2cm	23	2	21		15	8	
>2cm	30	13	17		17	13	
TNM stage				0.032*			0.389
I	19	2	17		10	9	
II–IV	34	13	21		22	12	
Grade				0.572			0.958
Well	25	8	17		15	10	
Moderate/poor	28	7	21		17	11	
Lymph node met-astasis				0.831			0.965
No	33	9	24		20	13	
Yes	20	6	14		12	8	

*p < 0.05, **p < 0.01. The value was statistically significant(highlighted in bold).

### Animals

This research protocol was approved by the Ethics Committee for Animal Research (No. 2016-067) in accordance with the Ethical Principal in Animal Experimentation by Guangzhou Medical University. The generation of HGF high expression transgenic mice (HGF-Tg, *n* = 50, female) and genotyping followed previously used protocols (20). C57BL/6 mice (Wt, *n* = 54, female) were purchased from Guangdong Experimental Animal Center. The experiments were performed under controlled conditions, including a 24-h light/dark cycle, and the mice were maintained in a room with a relative humidity level of 30%–50% and a temperature of 18–25°C. The mice were fed standard mouse chow.

### 4NQO-Induced Tumorigenesis

**Figure 1:**
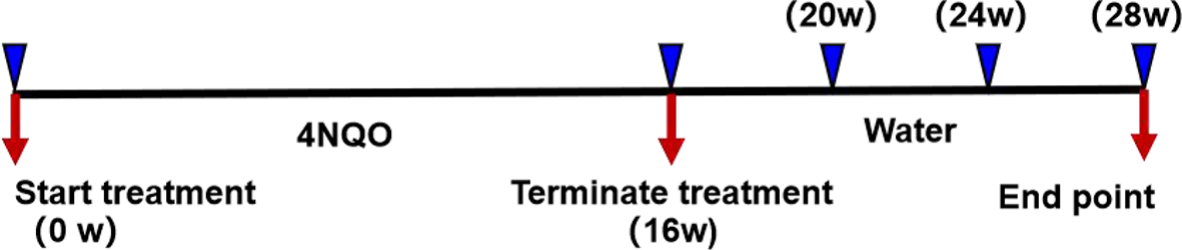
Representation of experimental design. After 4NQO treatment for 16 weeks, mice were sacrificed at 16, 20, 24, and 28 weeks.

The carcinogen 4NQO (Sigma-Aldrich, St. Louis, MO, USA) was prepared at a concentration of 5 mg/ml in propylene glycol and stored at 4°C. The 4NQO stock solution was diluted to a concentration of 50 µg/ml in drinking water, and the water was replaced once weekly. Six- to eight-week-old HGF-Tg mice and Wt mice were randomly divided into a control group (*n* = 18) and a 4NQO group (*n* = 86), respectively. The experimental group was drinking water containing 4NQO continually for 16 weeks, whereas the control group contained no 4NQO and the same volume of propylene glycol. Mice were sacrificed after 16, 20, or 24 weeks or when the body weight loss was ≥1/3 (21). The death time of naturally dead mice was recorded throughout the experiment, and the remaining surviving mice were sacrificed at 28 weeks. All mice were carefully inspected daily and weighed weekly. The experimental process is shown in **Figure 1**.

### Clinical Analysis

The mouse tongue was collected and photographed by stereo light microscopy. The number of tongue tumors larger than 0.5 mm was recorded. The volume of exophytic tumors was calculated using the formula 0.5 × (L × S^2^), where *L* is the longest diameter and *S* is the shortest diameter. The tumor volume of each mouse was the sum of all tumor volumes (22). To reduce the experimental error, photographing and recording the dimensions of the tumor were performed by Xiaoxi He.

### Histopathological Analysis

The tongues were fixed in 10% formalin, embedded in paraffin, sectioned into 4-µm sections, and stained with hematoxylin and eosin (H&E). The sections were examined by two pathologists at our institution and classified as follows: (a) normal epithelia, (b) mild dysplasia, (c) moderate dysplasia, (d) severe dysplasia, and (e) oral cancer, including extraneous papillary tumors (23, 24).

### Immunohistochemistry

IHC was performed using the streptavidin-peroxidase (SP) method as previously reported (25). In short, after fixation with 10% formalin for at least 24 h, the paraffin-embedded tissue was sliced into 4-µm sections. The sections were deparaffinized and rehydrated with xylene and ethanol. Antigen recovery was performed in a pressure cooker for 10 min in citrate buffer (pH = 6.0). Then, endogenous peroxidase activity was blocked with 3% H_2_O_2_ for 15 min. After blocking with 5% bovine serum albumin (BSA) for 30 min, sections was incubated overnight at 4℃ with the following antibodies: HGF for mouse paraffin sections (1:100, Bioss, #bs-1025R, Beijing, China), HGF for human paraffin sections (1:200, Proteintech, #26881-1-AP, Chicago, IL, United States), Met for mouse paraffin sections (1:100, Bioss, #bs-0668R, Beijing, China), Met for human paraffin sections (1:200, Proteintech, #25869-1-AP, Chicago, IL, United States), and PCNA (1:100, Proteintech, #24036-1-AP, Chicago, IL, United States). The tissue sections were then washed, incubated with the secondary antibody labeled with horseradish peroxidase (1:100, Beyotime, #A0208, Shanghai, China) for 1 h at room temperature and developed using DAB (SignalStain^®^ DAB Substrate Kit, #8059, Cell Signaling, Boston, MA, USA). Finally, the nuclei were counterstained with hematoxylin. Negative controls by omitting the primary antibody and positive controls were done for all tissues.

Each section was independently evaluated by two pathologists. The average percentage of PCNA-positive cells was measured in ≥5 random fields at ×400 magnification in each section. Only distinct nuclear staining was considered positive. The images were obtained using a Nikon microscope, and Image-Pro Plus software was used to count the number of positive cells. The HGF and c-Met staining scores were multiplied by the staining intensity (0, no staining; 1, light yellow; 2, moderate yellow brown; 3, strong brown staining) and the proportion of positive cells (0, negative; 1, 1%–25%; 2, 26%–50%; 3, >50%) (17, 26). The scores ranged from 0 to 9.

### TUNEL Assay

TUNEL staining was performed on 4-µm sections according to the manufacturer’s protocol (KeyGEN, #KGA704, Beijing, China). In brief, the sections were deparaffinized followed by antigen retrieval, labeled with biotin-dUTP for 30 min at room temperature, and incubated with streptavidin-HRP solution for 30 min in the dark at room temperature. The sections were then washed and developed using DAB. Finally, the nucleus was dyed with hematoxylin. A nucleus with positive expression appeared brownish yellow, and a nucleus with negative expression appeared blue. Apoptosis was evaluated by counting the proportion of TUNEL-positive cells in ≥5 images randomly selected at ×400 magnification.

### RNA Sequencing

High-throughput sequencing was conducted by Origingene (Shanghai, China). In short, Trizol reagent (Invitrogen, Carlsbad, CA, USA) was used to extract total RNA from the tongue tumor samples of Wt and HGF-Tg mice at 24 weeks (*n* = 4). The RNA amount and purity were detected using a NanoDrop spectrophotometer (NanoDrop 2000, Wilmington, DE, United States), and the RNA concentration was determined using a Qubit. The RNA integrity was assessed by Agilent 2100 with RIN number >7.0. RNA-Seq libraries were prepared using a TruSeq™ RNA sample preparation Kit (Illumina) and sequenced using an Illumina HiSeq X-Ten (LC Bio, China) following the vendor’s recommended protocol. Cutadapt was used to remove the reads that contained adaptor contamination, low-quality bases, and undetermined bases. Then, sequence quality was verified using FastQC (http://www.bioinformatics.babraham.ac.uk/projects/fastqc/). RNA-Seq read data were mapped to the reference genome of mouse species (GRCm38) using HISAT2 software. The mapped reads of each sample were assembled using StringTie (27). Then, the transcriptomes from all samples were merged to reconstruct a comprehensive transcriptome using Perl scripts. After the final transcriptome was generated, StringTie and Ballgown were used to estimate the expression levels of all transcripts (27, 28). Differentially expressed genes (DEGs) were examined using the R/Bioconductor package edgeR and established based on a log2-fold change and *p*-value (**|**log2(FC)**|** ≥ 1.00; *p < 0*.05). Gene set pathway analysis was performed using DAVID bioinformatics tools. Kyoto Encyclopedia of Genes and Genomes (KEGG) pathway analysis was performed by setting all the KEGG pathway genes as background genes. The enrichment *p*-value calculation was performed with the Fisher’s exact test.

### Real-Time Quantitative PCR Analysis

Eleven DEGs involved in the PI3K-AKT and MAPK pathways were verified by Q-PCR. Total RNA was extracted using an RNA Extraction Kit (19221ES50, YEASEN, China). The quantity and quality of extracted RNA were analyzed with 1.8 to 2.0 of the OD260/280 using a spectrophotometer (Nanodrop 2000, Thermo Fisher Scientific, USA). Double-stranded cDNA was synthesized using a PrimeScript RT kit (Cat No. AG11706, ACCURATE BIOLOGY, China) according to the manufacturer’s instructions. Q-PCR was performed using SYBR Premix ExTaq™ II (Cat No. AG11718, ACCURATE BIOLOGY, China). The 2^−δδCT^ method was used to quantify the relative expression of each mRNA, and GAPDH served as an internal reference gene. All experiments were conducted in triplicate. The primer sequences are shown in **Supplementary Table 1**.

### Statistical Analysis

The data were analyzed using SPSS 17.0 software and GraphPad Prism 6.0. According to the IHC score of HGF and c-Met expression, OSCC patients were divided into a low group (0–4) and a high group (>4). The chi-square test was used to evaluate the significance of the association between HGF expression and clinicopathological parameters. Kaplan–Meier analysis and log-rank tests were performed to estimate cumulative survival. Statistical significance among two or more groups was tested by unpaired Student’s *t*-test and Mann–Whitney test. *p < 0*.05 was considered a significant difference.

## Results

### HGF and c-Met expression in OSCC Specimens

**Figure 2:**
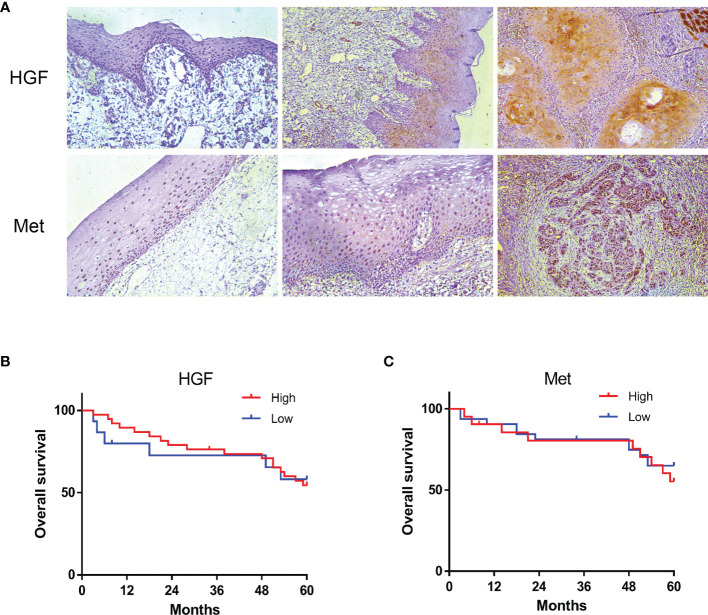
HGF and c-Met immunostaining in human normal oral mucosa, paracancerous tissue, and OSCC tissue. ×200 **(A)**. The 5-year overall survival rate of OSCC patients (*p* > 0.05). Kaplan–Meier analysis **(B, C)**.

Tissue samples from 53 OSCC patients were immunohis-tochemically stained for HGF and c-Met expression. HGF protein was expressed in cytoplasm, cell membrane, and nucleus, and Met protein was mainly expressed in cytoplasm and membrane. Immunostaining for HGF and c-Met was negative or extremely weak in the normal oral mucosa but positive in 92.5% (49/53) and 64% (34/53) of OSCC samples, respectively. Compared with adjacent nontumor tissues, HGF and c-Met expression were relatively higher in tumor tissues (**Figure 2A**).

To further understand the role of HGF and c-Met in OSCC progression, we analyzed the associations between tissue HGF and c-Met levels and clinicopathological features. As shown in **Table 1**, high HGF expression was correlated with smaller tumor size (*p < 0*.006) and *inferior* TNM stage (*p < 0*.032), and no significant relationship was found between HGF expression and sex, age, grade, lymph node metastasis, or any other clinicopathological parameters. However, no significant correlation was noted between c-Met expression levels and clinical parameters. In addition, HGF and c-Met expression was not significantly associated with overall survival (OS) in patients with OSCC (**Figures 2B, C**).

### Expression of HGF and Its Receptor c-Met in Tumors Induced by 4NQO

**Figure 3:**
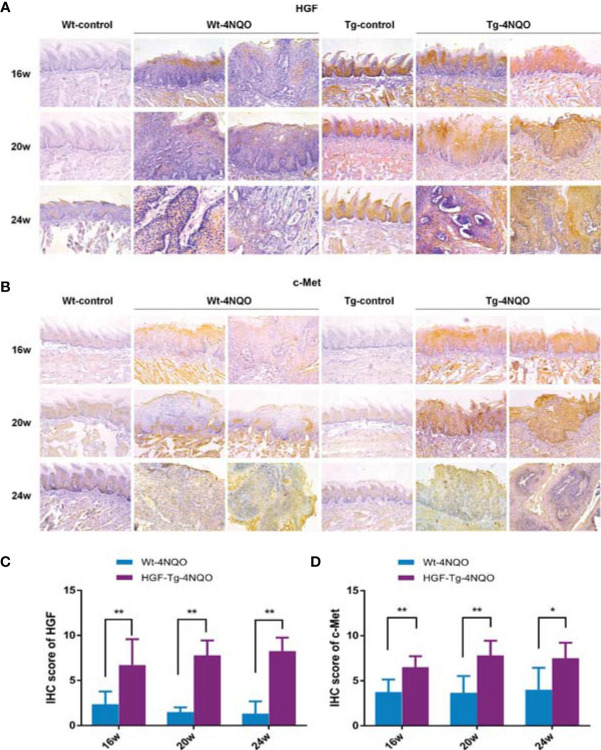
HGF and c-Met immunostaining in mouse tongue lesions of different groups. Representative IHC images of HGF **(A)** and c-Met **(B)**.×200. IHC scores of HGF **(C)** and c-Met **(D)**. 4NQO group, 16 w: *n* = 8; 20 w: *n* = 7 (Tg) and *n* = 8 (Wt); 24 w: *n* = 6 (Tg) and *n* = 9 (Wt). Mean ± SEM. Mann–Whitney test. *p < 0.05, **p < 0.01.

Similar to human OSCC tissues, HGF was not expressed or was weakly expressed in normal oral mucosa of the Wt-control group but was upregulated in epithelial dysplasia and tumor tissues in the Wt-4NQO group (**Figure 3A**, left panels). As a unique receptor of HGF, we evaluated c-Met staining by immunohistochemistry in different groups. We found that c-Met expression was also upregulated during carcinogenesis but was slightly positively expressed in the normal tongue epithelium (**Figure 3B**, left panels). From 16 to 24 weeks, the epithelial lesions of 4NQO-treated mice gradually deteriorated from mild dysplasia to invasive carcinoma. In this process, we found that HGF expression was gradually weakened in the Wt-4NQO group, but the result was not statistically significant (data not shown). In addition, we did not observe a difference in c-Met expression between dysplasia and tumor tissue in Wt mice (**Figure 3B**, left panels).

Compared with Wt mice, HGF staining was significantly stronger in normal epithelial tissue of Tg mice (**Figure 3A**). HGF was mainly overexpressed in normal oral epithelium of HGF-Tg mice, while it was upregulated in both epithelium and connective tissues of 4NQO-treated Tg and Wt mice (**Figure 3A**). However, c-Met was slightly positively expressed in the normal epithelium of both Wt and HGF-Tg mice (**Figure 3B**). In an oral carcinogenesis model induced by 4NQO, HGF and c-Met proteins maintained high levels compared to Wt mice from 16 to 24 weeks (**Figures 3C, D**).

### HGF-Tg Mice Were Less Susceptible to Oral Cancer Induced by 4NQO

**Figure 4:**
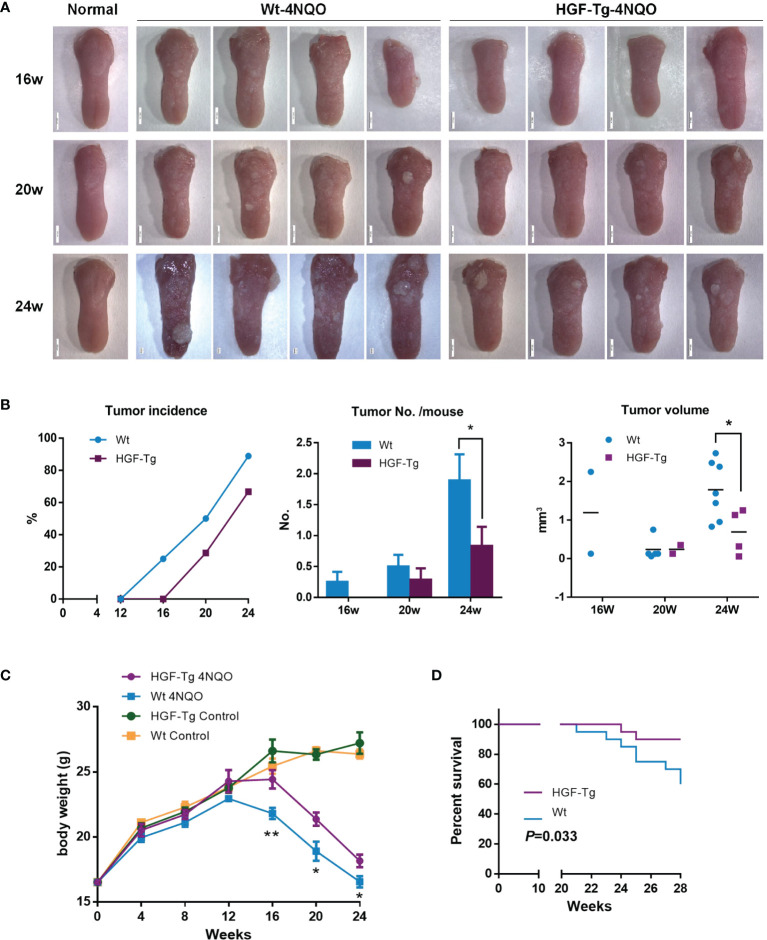
Induction of mouse tongue tumorigenesis by 4NQO. Representative images of mouse tongue **(A)**. T*umor* incidence, numbers, and volume in mice. Student’s *t*-test **(B)**. The body weights of control and 4NQO-treated mice. Wt mice exhibited significantly reduced body weight compared with HGF-Tg mice treated with 4NQO. Student’s *t*-test **(D)**. Kaplan–Meier survival analysis **(E)**. 4NQO group, 16 w: *n* = 8; 20 w: *n* = 7 (Tg) and *n* = 8 (Wt); 24 w: *n* = 6 (Tg) and *n* = 9 (Wt); 28 w: *n* = 20. Control group, *n* = 3 (16/20/24 w). Mean ± SEM. *p < 0.05, **p < 0.01.

As shown in **Figure 4A**, the surface of normal tongue mucosa was pink and soft, the lingual papillae were evenly distributed, and there were no white patches or tumor formation. After drinking 4NQO water for 16 weeks, the lingual papilla disappeared regionally, and white patches of different sizes emerged. Over time, the tongue surface showed several leukoplakia and developed into papillary tumors.

Wt mice began to develop tumors 12–16 weeks after 4NQO induction, whereas HGF-Tg mice started to develop tumors at 16–20 weeks. The incidence of tumors in Tg mice was always lower than that in Wt mice (**Figure 4B**, left panels). The tumor number on the tongue surface of HGF-Tg mice was significantly less than that in Wt mice (**Figure 4B**, middle). At week 24, most 4NQO-treated mice showed typical pathological features of tongue tumors, with the tumor volume of the HGF-Tg group being significantly smaller (**Figure 4B**, right panels).

Throughout the 28-week experimental period, we noticed that both HGF-Tg mice and Wt mice exposed to 4NQO began to lose weight at 12 weeks, but the weight of Wt mice decreased more seriously than HGF-Tg mice (**Figure 4C**). In addition, the survival rate of HGF-Tg mice was significantly greater than that of Wt mice (*p < 0*.033, **Figure 4D**).

**Figure 5:**
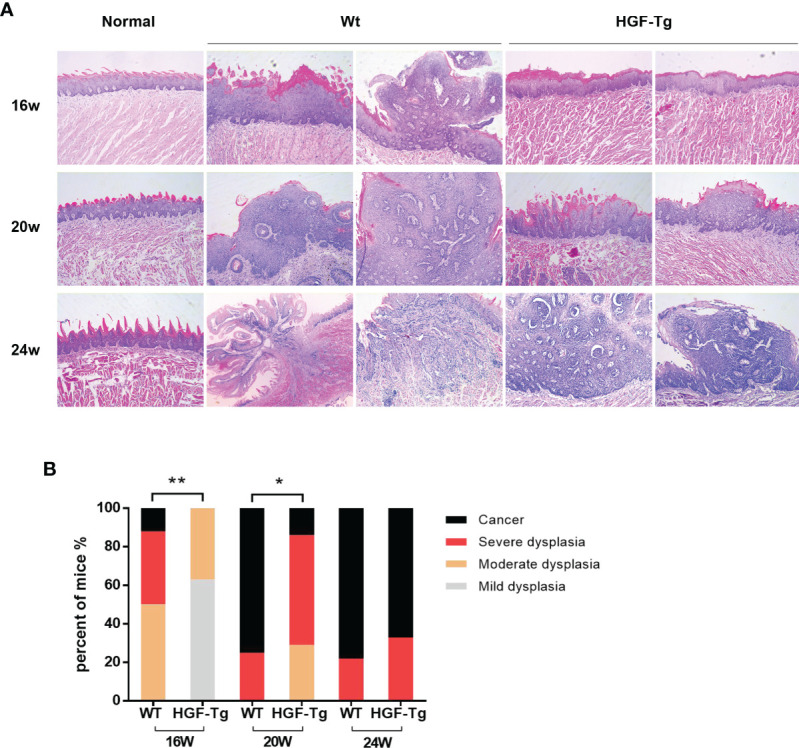
Representative HE sections of mice tongue, including dysplastic tissues and carcinoma. With the exception of the left image of Wt mice at 24 weeks, which was magnified at ×50, the remaining images were magnified at ×100 **(A)**. Quantification of histopathological grading of tongue. Chi-square test **(B)**. 4NQO group, 16 w: *n* = 8; 20 w: *n* = 7 (Tg) and *n* = 8 (Wt); 24 w: *n* = 6 (Tg) and *n* = 9 (Wt). *p < 0.05, **p < 0.01.

H&E staining directly revealed dynamic histopathological changes in carcinogenesis induced by 4NQO compared with normal epithelial tissue (**Figure 5A**). At the 16th week, the tongue epithelium of Tg mice showed mild and moderate dysplasia, while the Wt mice had papillary tumors. At 24 weeks, invasive tumors were noted in the Wt group, but the basement membrane of the tumor in transgenic mice remained intact. The grade of dysplasia lesions and neoplasm incidence in HGF-Tg mice treated with 4NQO were significantly lower than those in Wt mice (*p < 0*.05, **Figure 5B**). Histopathological analysis further confirmed that HGF-Tg mice were less sensitive to 4NQO-induced oral tumorigenesis.

### Overexpression of HGF Affects Cellular Proliferation and Apoptosis in Tumor Tissue

**Figure 6:**
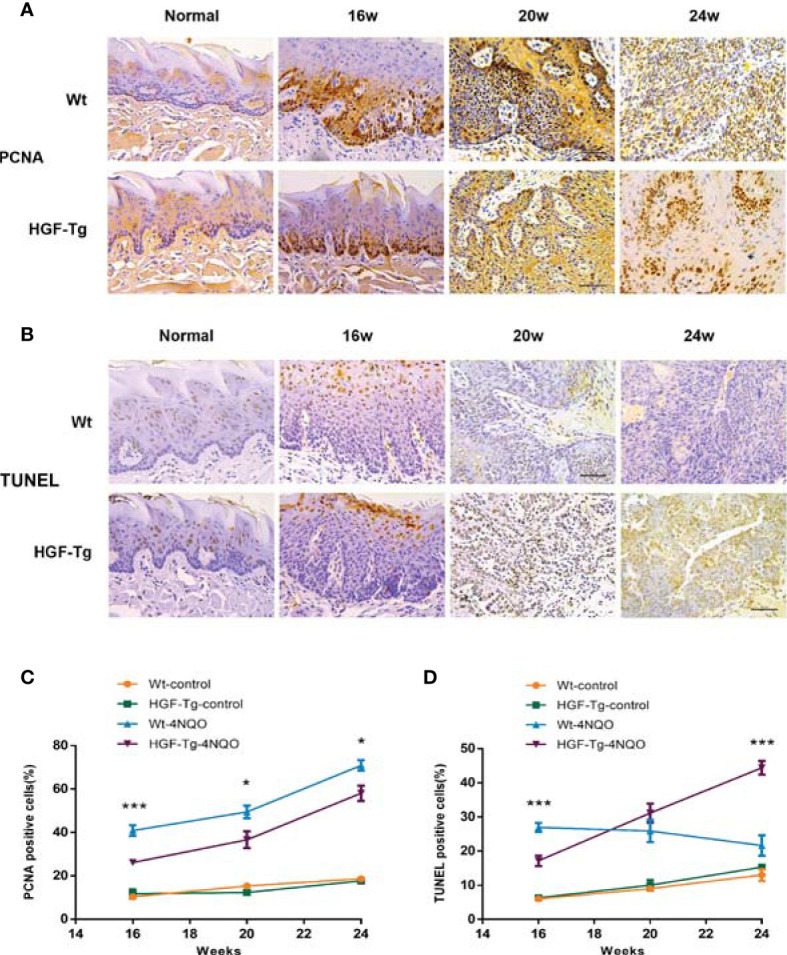
Proliferation and apoptosis in tongue lesions. Representative IHC PCNA staining images **(A)** and percentage of PCNA-positive cells **(C)**. Representative TUNEL staining images **(B)** and percentage of TUNEL-positive cells **(D)**. All pictures were obtained at ×400 magnification. 4NQO group, 16 w: *n* = 8; 20 w: *n* = 7 (Tg) and *n* = 8 (Wt); 24 w: *n* = 6 (Tg) and *n* = 9 (Wt). Control group, *n* = 3 (16/20/24 w). Mean ± SEM. Student’s *t*-test. *p < 0.05, ***p < 0.001.

To further observe the pathological changes of tongue lesions of Tg mice and Wt mice, we used IHC to detect PCNA and TUNEL analysis for apoptosis (**Figure 6**). During 4NQO-induced carcinogenesis, PCNA-positive cells increased continuously in the process of transforming from mild dysplasia to cancerous tissue. Moreover, the proportion of proliferating cells in Wt mice was relatively greater than that in Tg mice treated with 4NQO from 16 to 24 weeks (*p < 0*.05) (**Figures 6A, C**), suggesting that HGF may inhibit cell proliferation during tumorigenesis.

Additionally, the number of apoptotic cells in lesions of 4NQO-treated Wt mice gradually declined from 16 to 24 weeks, whereas that in Tg mice increased during the experiment. At 16 weeks, the number of TUNEL-positive cells in the Tg-4NQO group was significantly lower than that in the Wt-4NQO group. However, at 24 weeks, the number was significantly greater than that in the Wt-4NQO group (**Figures 6B, D**).

### Downregulation of MAPK and PI3K-AKT Signaling Potentially Affects Tumorigenesis in HGF-Tg Mice

**Figure 7:**
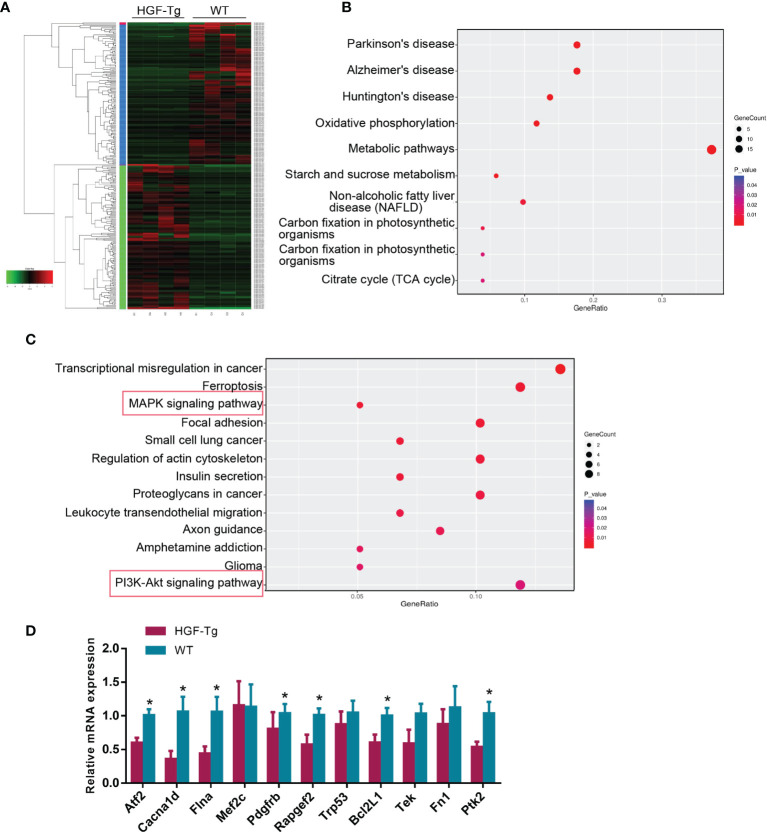
RNA sequencing of tongue t*umor* tissues of Wt and Tg mice at 24 weeks. Heatmap of 277 differentially expressed genes **(A)**. Bubble plot represents the top 10 KEGG pathways with 140 upregulated DEGs **(B)** and top 13 KEGG pathways with 137 downregulated DEGs **(C)**. Q-PCR data of DEGs enriched in the MAPK and PI3K-AKT pathway **(D)**. Mean ± SEM. *n* = 4. Student’s *t*-test. *p < 0.05.

To further explore the downstream molecular mechanism of HGF overexpression, we performed RNA-seq using tongue tumor tissues harvested from Wt and HGF-Tg mice. As indicated in the heatmap generated for DE genes (**Figure 7A**), 140 upregulated DEGs and 137 downregulated DEGs were identified from our RNA-seq data. To determine pathways regulated by HGF overexpression, KEGG analysis was performed, and the data are presented as a bubble plot. KEGG analysis showed that the upregulated DEGs were generally enriched in signaling pathways, such as oxidative phosphorylation (*p* = 0.00064); metabolic pathways (*p* = 0.00084); starch and sucrose metabolism (*p = 0*.001); and the citrate cycle (TCA cycle) (*p = 0*.019) (**Figure 7B**). The downregulated DEGs were significantly enriched in MAPK signaling (*p = 0*.0055) and the PI3K-AKT pathway (*p* = 0.018) (**Figure 7C**). We further analyzed the expression pattern of DEGs associated with MAPK and PI3K-Akt signaling that are most related to proliferation and apoptosis. Q-PCR results showed that the mRNA expression of DEGs (*Atf2, Cacna1d, Flna, Pdgfrb, Rapgef2, Bcl2L1*, and *Ptk2*) in HGF-Tg mice was significantly decreased compared with that in Wt mice, which was consistent with the RNA-seq data (**Figure 7D**). Therefore, our data suggested that inhibition of the MAPK and PI3K-AKT pathways may play a role in the progression of tongue tumor of HGF-Tg mice.

## Discussion

It has been extensively reported that HGF and Met overexpression, gene amplification, or mutation is found in numerous tumors (12, 29). Similarly, in this study, we showed that HGF and c-Met were upregulated in OSCC and adjacent nontumor tissues. In addition, compared with normal epithelium, HGF and its receptor c-Met were positively expressed in dysplastic and tumor tissues in an oral cancer model of Wt mice. Moreover, we investigated the correlation between HGF/c-Met signaling and HNSCC, and its association with survival of HNSCC patients using data obtained from The Cancer Genome Atlas (TCGA) (**Supplementary Materials**). It showed that HGF expression increased in HNSCC patients with no statistical significance (**Supplementary Figure A,a**), but c-Met was significantly overexpressed compared with normal counterpart (**Supplementary Figure B,a**). The data suggested that HGF/c-Met signaling is closely correlated with the progression of OSCC.

In addition, we found that high HGF expression was associated with smaller tumor size and *inferior* TNM stage but was not related to other clinical parameters or OS in OSCC patients. TCGA data revealed that HGF expression was not correlated with prognosis of HNSCC patients (**Supplementary Figure A,b**). However, Uchida et al. reported no association between HGF expression in serum and oral cancer tissues and tumor size, lymph node status, metastasis and prognosis, although the serum levels of HGF in OSCC patients were significantly increased compared with those in healthy people (30). Chen et al. reported that only HGF expression in the tumor invasion front, not the tumor center, was correlated with lymph node status and the clinical stage of OSCC in Taiwan (31). Thus, a definite conclusion about the correlation between HGF expression level and clinical parameters and prognosis is lacking, and more clinical samples are needed.

A meta-analysis showed that c-Met overexpression in HNSCC was significantly correlated with poor OS and regional lymph node metastases (32). Another systematic review suggested that c-Met overexpression as assessed by immunohistochemistry could represent a promising prognostic biomarker of HNSCC, but the best scoring method remains to be determined (33). However, consistent with the findings of Sun et al., our study showed no significant correlation between c-Met expression in OSCC and clinicopathological variables. Moreover, according to TCGA data, there was no association between c-Met expression and OS of HNSCC patients (**Supplementary Figure B,b**).

In oral cancer models, HGF-Tg mice had a smaller tumor number and tumor volume and a higher survival rate than Wt mice. We observed greater HGF expression in dysplastic tissue compared with tumor tissue from the Wt-4NQO group, but the data were not statistically significant. At 16, 20, and 24 weeks, the pathological changes in the tongue in Wt mice were more serious than those in HGF-Tg mice. Based on our clinical samples and *in vivo* evidence, our research supports the view that HGF expression is associated with tumor size and the stage of oral cancer, and HGF overexpression can slow tongue tumor induction by 4NQO.

Intriguingly, we discovered that activation of the HGF/c-Met signaling pathway can inhibit the occurrence of oral cancer, although aberrant HGF and c-Met expression in tumor tissues is considered to be a promoting factor for the development of various types of cancer (34). In addition, Liu et al. reported that HGF can suppress hepatocarcinogenesis induced by diethylnitrosamine, indicating that HGF has different effects on the growth of normal hepatocytes and tumor hepatocytes *in vivo* (35). Therefore, combined with the latest relevant research results, we believe that the HGF/c-Met signaling pathway plays a two-way regulatory role in different cell types or under different conditions. Abnormal expression of the HGF/c-Met pathway in tumor tissues can promote tumor progression, whereas HGF overexpression in normal tissues can inhibit tumorigenesis.

As a multifunctional growth factor, HGF plays an important role in cell proliferation, survival, motility, and morphogenesis (36). In this study, PCNA immunostaining was used to assess the proliferation status of the lesions. The higher the cell proliferation rate, the higher the risk of cell mutation and malignant transformation (37). Findings showed that the HGF-Tg mice had fewer PCNA-positive cells than the Wt mice treated with 4NQO. Similarly, Tajima et al. demonstrated that HGF has a strong antiproliferative effect in various tumor cell lines, including HepG2 hepatocellular carcinoma cells, KB squamous carcinoma cells, and B6/F1 melanoma cells, *in vitro* and *in vivo* (38). In contrast, Knowles et al. showed that HGF promotes cell growth in HNSCC cell lines (39). Thus, we hypothesized that HGF may have multifunctional effects on cell growth in different cell types. Although the expression of PCNA is considered a useful marker in evaluating the malignant degree and progression of tumors (40), due to its long half-life (approximately 20 h), involvement in other cellular processes such as DNA repair, epitopic differences and other factors, and higher values of PCNA positivity might often be detected (41, 42); thus, there is a need to add other cell proliferation markers in subsequent studies.

During 4NQO-induced oral carcinogenesis, HGF overexpression promotes cell apoptosis based on TUNEL results. Conner et al. discovered that HGF inhibits hepatocarcinogenesis in transgenic mice and induces apoptosis in transformed rat liver epithelial cells (43). Furthermore, it has been reported that JNK1 induction, protein kinase C, and matrix metalloproteinase induction are involved in the apoptotic process induced by HGF (44). Consistent with our findings, Tulasne et al. revealed that the HGF/c-Met axis has proapoptotic and antiapoptotic properties in different cell types or stress conditions, which is important for maintaining the cell survival/apoptosis balance (45). They concluded that although HGF/c-Met signaling is involved in invasive growth and the antiapoptotic response in most tumor cell types, it can induce apoptosis and prevent malignant transformation in some cases, such as in some transformed cell lines.

4NQO is a water-soluble chemical carcinogen that causes DNA mutations and DNA double-strand breaks while increasing reactive oxygen species (ROS) production and forming large DNA adducts (46). An increase in intracellular ROS can affect cell apoptosis, survival, and differentiation *via* MAPK signaling consisting of ERK, JNK, and p38 (47, 48). Choi et al. demonstrated that HGF overexpression can reduce the level of intracellular ROS and inhibit H_2_O_2_-induced apoptosis (3). Similarly, previous studies have reported that HGF can protect vascular endothelial cells from oxidative damage induced by hypoxia/reoxygenation (49, 50). In addition, HGF can reduce H_2_O_2_-induced apoptosis of neural progenitor cells derived from human embryonic stem cells (51). These studies indicated that HGF regulates oxidative stress. In our study, RNA-Seq data showed that upregulated DEGs are highly correlated with oxidative and metabolic signaling.

Moreover, HGF overexpression in normal tissues can inhibit cell proliferation and accelerate apoptosis in the progression of oral cancer potentially by downregulating MAPK and PI3K-AKT according to KEGG enrichment analysis. As important intracellular signal transduction pathways, the two signaling pathways have highly similar physiological functions and jointly regulate cell growth, development, differentiation, and apoptosis (52). Although studies have shown that the HGF/c-Met pathway is abnormally activated in cancer and can promote tumor proliferation, invasion, metastasis, and angiogenesis by stimulating the PI3K/AKT, Ras/MAPK, JAK/STAT, SRC, Wnt/β-catenin, and other signaling pathways (10, 34), our study suggested that HGF/c-Met signaling may inhibit the development of tumor cells transformed from normal cells with high expression of HGF through the regulation of these two pathways, and thus provides a direction for the mechanism of our future research.

Nowadays, HGF/c-Met signaling has been recognized as a promising cancer therapeutic target, and many types of inhibitors have been developed to eliminate the activation of this pathway (53). However, the therapeutic effect of a large number of HGF/c-Met targeted drugs is not satisfactory and faces great challenges at present (54). This might be due to the signal pathway having an anticancer effect in the malignant stage of normal cells, which requires the attention of researchers.

In this study, we used HGF-overexpression transgenic mice (globally expressed) and tongue tumor models induced by 4NQO to explore the role of HGF/c-Met signaling in oral tumorigenesis. A limitation of the research is that we mainly focused on the HGF protein secreted by oral epithelial tissues, but HGF was upregulated in both epithelium and stromal tissue of HGF-Tg and Wt mice with 4NQO treatment. Since HGF is expressed in multiple cells, multiple tissues, and multiple organs in Tg mice, the function and role of HGF produced by other cells or tissues could not be confirmed, which might also affect the malignant transformation of epithelial tissues. A previous study found that HGF is commonly secreted by stromal cells and then activates the Met receptors in tumor cells by paracrine in the tumor microenvironment, while HGF autocrine by tumor cells rarely occurs (10). Therefore, the next plan was to adopt the Cre-loxP methodology to generate transgenic mice with overexpression of specific cell or tissue expression, and separately culture epithelial cells and stromal cells, such as fibroblasts, in order to study its function and mechanism more accurately.

## Conclusion

In conclusion, our study found that HGF/c-Met signaling was closely associated with the occurrence and development of OSCC, and the overexpression of HGF in normal oral epithelial tissue can inhibit 4NQO-induced tumorigenesis probably by reducing cell proliferation and accelerating apoptosis *via* MAPK and PI3K-AKT signaling; however, its function and mechanism of action require further experiments.

## Data Availability Statement

All raw RNA-sequencing data can be accessed from NCBI SRA database (SRA accession: PRJNA779172).

## Ethics Statement

The studies involving human participants were reviewed and approved by the Human Ethics Research Committee of Stomatological Hospital Affiliated to Guangzhou Medical University (KY2019025). The patients/participants provided their written informed consent to participate in this study. The animal study was reviewed and approved by the Ethics Committee for Animal Research (No.2016-067) in accordance with the Ethical Principal in Animal Experimentation by Guangzhou Medical University.

## Author Contributions

XW, XH, and SC contributed to the conception and design of the study. SC and XC collected OSCC specimens and patient’s clinical data. XH, YT, XZ, ZW, and WL performed the experiments. XH, YT, LY, and SC performed the statistical analysis. XH wrote the first draft of the manuscript. XH, SC, LW, and XW critically revised the manuscript. All authors contributed to the manuscript revision and read and approved the submitted version.

## Funding

This work was supported by the Medical Scientific Research Foundation of Guangdong Province, China (grant numbers A2016223) and by the National Natural Science Foundation of China (grant numbers 31801152).

## Conflict of Interest

The authors declare that the research was conducted in the absence of any commercial or financial relationships that could be construed as a potential conflict of interest.

## Publisher’s Note

All claims expressed in this article are solely those of the authors and do not necessarily represent those of their affiliated organizations, or those of the publisher, the editors and the reviewers. Any product that may be evaluated in this article, or claim that may be made by its manufacturer, is not guaranteed or endorsed by the publisher.
