# Supplementary material for: Hepatocyte Growth Factor Overexpression Slows the Progression of 4NQO-Induced Oral Tumorigenesis
Source: Front Oncol. 2021 Dec 14;11:756479. doi: 10.3389/fonc.2021.756479 (PMC8712676; doi:10.3389/fonc.2021.756479)
Supplement: Supplementary file 1 [file DataSheet_1.docx]

**Supplementary materials**

**Bioinformatics analysis of HGF and Met from The Cancer Genome Atlas (TCGA) database**

The RNA sequencing and clinical data of 519 HNSCC patients were obtained from TCGA database (https://www.cancer.gov/tcga.). The correlation between HGF and c-Met expression and HNSCC were analyzed by the Gene Expression Profiling Interactive Analysis 2.0 (GEPIA2, <http://gepia2.cancer-pku.cn.).>

**Figures lends.**

Fig. 1 Association of HGF and Met overexpression with HNSCC patients in TCGA database. HGF expression increased in HNSCC patients compared with normal counterpart, but there was no statistical significance (*P*>0.05, A-a), and HGF expression was not significantly associated with overall survival (OS) of HNSCC patients (*P*>0.05, A-b). Met were significantly overexpressed in HNSCC (*P*<0.05, B-a), but had no correlation with OS of HNSCC patients (*P*>0.05, B-b). **P*<0.05.
