# Supplementary figures and images for: Hepatocyte Growth Factor Overexpression Slows the Progression of 4NQO-Induced Oral Tumorigenesis
Source: Front Oncol. 2021 Dec 14;11:756479. doi: 10.3389/fonc.2021.756479 (PMC8712676; doi:10.3389/fonc.2021.756479)

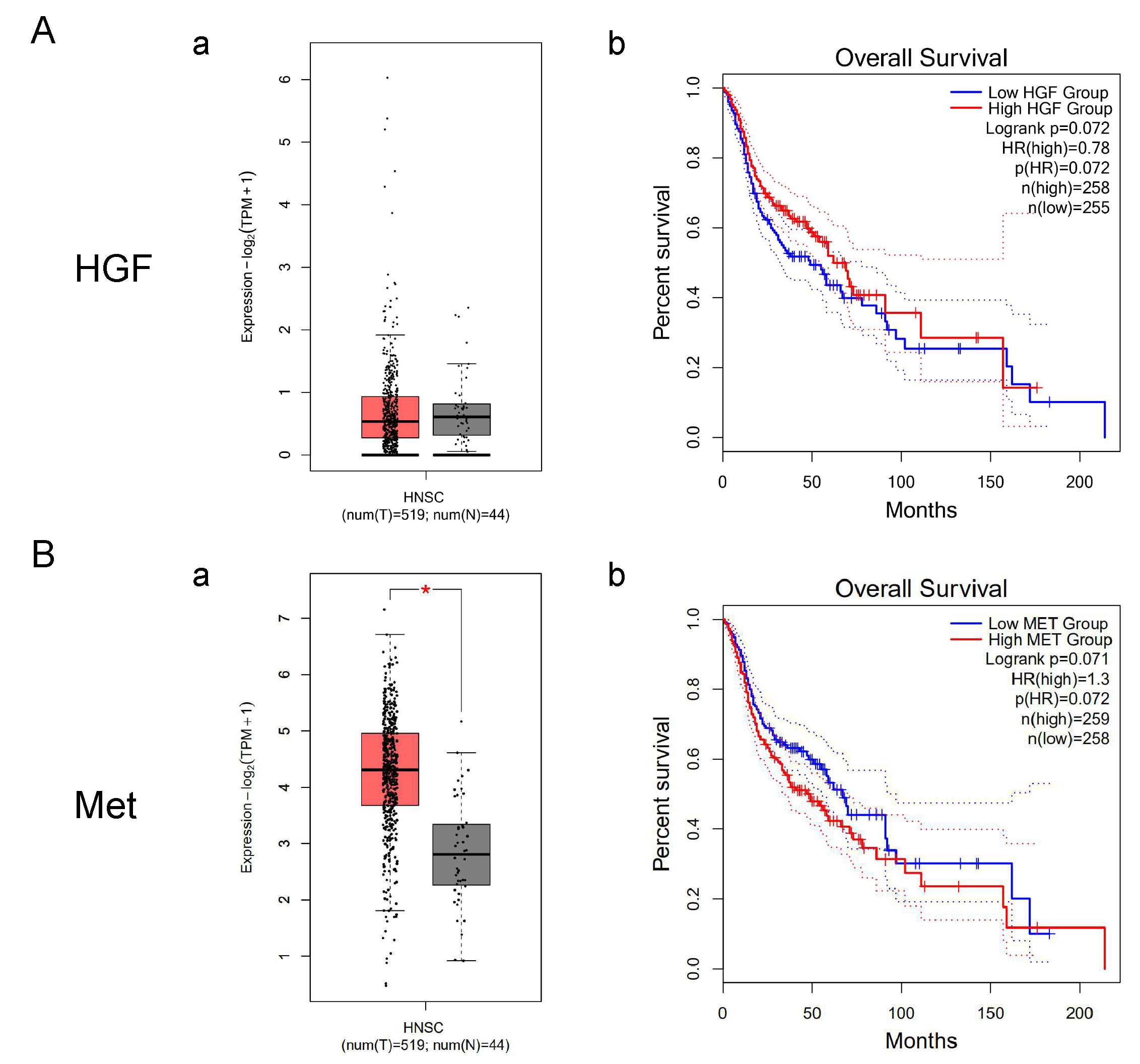

Supplement: Supplementary file 2 [file Image_1.tif]
