# Supplementary material for: Hepatocyte Growth Factor Overexpression Slows the Progression of 4NQO-Induced Oral Tumorigenesis
Source: Front Oncol. 2021 Dec 14;11:756479. doi: 10.3389/fonc.2021.756479 (PMC8712676; doi:10.3389/fonc.2021.756479)
Supplement: Supplementary file 3 [file Table_1.doc]

| **Gene Name** | **Forward primer (5'->3')** | **Reverse primer (5'->3')** |
| --- | --- | --- |
| Atf2 | ATGGCAGTGGATTGGTTAGG | AGTTGTGTGAGCTGGAGAAG |
| Bcl2l1 | GGCGGCTGGGACACTTT | TCAGGAACCAGCGGTTGAAG |
| Pdgfrb | AGGAGTGATACCAGCTTTAGTCC | CCGAGCAGGTCAGAACAAAGG |
| Ptk2 | TTAGGCGATCCTATTGGGAGATG | TTCTTAGTGTTTTGGCCTTGACA |
| Tek | CTGGAGGTTACTCAAGATGTGAC | TCCGTATCCTTATAGCCTGTCC |
| Fn1 | ACCCGTTTTCATCCAACAAGAG | CGGTATCCAGACACCACACTATCA |
| Cacna1d | GCTTACGTTAGGAATGGATGGAA | GAAGTGGTCTTAACACTCGGAAG |
| Mef2c | GTCAGTTGGGAGCTTGCACTA | CGGTCTCTAGGAGGAGAAACA |
| Rapgef2 | GAGACGGCGGTTGACTCTG | TCCTGTCTATCGGGTCCTTCT |
| Trp53 | CCCCTGTCATCTTTTGTCCCT | AGCTGGCAGAATAGCTTATTGAG |
| Flna | CATCCCTCGTAGCCCCTACA | CCTTGGGACCCTTTACAGTGA |

**Supplementary Table 1. The primer sequences of eleven differentially expressed genes**
